# Association of circulating adipokine concentrations with indices of adiposity and sex in healthy, adult client owned cats

**DOI:** 10.1186/s12917-019-2080-9

**Published:** 2019-09-18

**Authors:** Maggie C. Williams, Chantal J. McMillan, Elisabeth R. Snead, Kanae Takada, Prasanth K. Chelikani

**Affiliations:** 1 Department of Veterinary Clinical and Diagnostic Sciences, Faculty of Veterinary Medicine, University of Calgary, 3330 Hospital Dr. NW, Calgary, AB Canada; 2 Department of Small Animal Clinical Sciences, Western College of Veterinary Medicine, University of Saskatchewan, 52 Campus Drive, Saskatoon, SK Canada; 3 Department of Production Animal Health, Faculty of Veterinary Medicine, University of Calgary, 3330 Hospital Dr. NW, Calgary, AB T2N 4N1 Canada

**Keywords:** Feline diabetes, Obesity, Sex, Adipokines, Omentin

## Abstract

**Background:**

Both diabetes mellitus (DM) and obesity are common in cats. The adipokines leptin, adiponectin, resistin and omentin are thought to have important roles in human obesity and glucose homeostasis; however, their functions in the pathophysiology of feline diabetes mellitus and obesity are poorly understood. We determined whether sexual dimorphism exists for circulating concentrations of these adipokines, whether they are associated with adiposity, and whether they correlate with basic indices of insulin sensitivity in cats. Healthy, client-owned male and female cats that were either ideal weight or obese were recruited into the study. Fasting blood glucose, fructosamine, cholesterol, triglycerides, insulin and plasma concentrations of adipokines were evaluated.

**Results:**

Obese cats had greater serum concentrations of glucose and triglycerides than ideal weight cats, but fructosamine and cholesterol concentrations did not differ between groups. Body weight and body mass index were greater in male than female cats, but circulating metabolite cocentrations were similar between sexes of both the ideal weight and obese groups. Plasma concentrations of insulin and leptin were greater in obese than ideal weight cats, with reciprocal reduction in adiponectin concentrations in obese cats; there were no sex differences in these hormones. Interestingly, plasma omentin concentrations were greater in male than female cats but with no differences between obese and ideal weight states.

**Conclusion:**

Together our findings suggest that rather than gender, body weight and adiposity are more important determinants of circulating concentrations of the adipokines leptin and adiponectin. On the contrary, the adipokine omentin is not affected by body weight or adiposity but instead exhibits sexual dimorphism in cats.

## Background

Diabetes mellitus (DM) is a common endocrinopathy affecting an estimated 0.3–1% of cats presenting to teaching hospitals and primary-care practices in the UK, Sweden and United States [1–5]. This condition resembles Type 2 DM (T2DM) in humans, with affected animals developing insulin resistance and failure of pancreatic β-cells to increase insulin production to maintain euglycemia [6, 7]. Though the exact causes are multifactorial, obesity is a major risk factor and has been linked to insulin resistance (IR), hyperinsulinemia, and altered hormone secretion from adipose tissue in cats [6, 8, 9]. Male cats have an increased prevalence of being overweight and developing DM when compared to female cats [5, 10–13]. However, little is known about the pathophysiological mechanisms that predispose males to DM or obesity.

The adipose tissue is a source of several hormones of which the adipokines - leptin, adiponectin, resistin, and omentin - play important roles in the pathophysiology of obesity and diabetes in humans. There is increasing interest in these adipokines in feline medicine. Leptin is a pro-inflammatory adipokine with serum concentrations that correlate positively with fat mass in both humans and cats [14–17]. Obese cats have higher circulating levels of leptin, and these decrease with weight loss [15, 16]. In health, leptin acts to help maintain energy homeostasis with increased levels promoting a decrease in appetite and increase in metabolic rate [8, 14]. However, a ‘leptin resistance’ is apparent in obese humans and rodent models, with a loss of responsiveness to leptin seen despite increased fat mass and serum leptin levels [18]. This dysregulation of leptin sensing and signaling may contribute to and perpetuate obesity in addition to promoting IR in cats [8, 18]. Contrary to leptin, adiponectin has anti-inflammatory properties and has been shown to be negatively correlated with fat mass in some [15, 19–22] but not all [17, 23] studies in cats. Further, adiponectin has been shown to be an insulin sensitizing hormone in mice [24, 25], and hypoadiponectinemia has been suggested as a contributing factor to the development of IR and DM in humans [14, 26]. We recently reported that adiponectin levels were decreased in diabetic cats when compared to both obese and lean non-diabetics [21]. Little research has been done to evaluate sex differences in circulating adipokine concentrations in cats. In humans, sex-specific differences in both adiponectin levels and their relationships to body composition have been shown [27]. Obese male humans have higher percentages of abdominal fat and this has been linked to an increased risk of DM. In neutered cats, however, no sex differences in fat distribution have been found, suggesting factors other than fat distribution are responsible for the increased prevalence of DM in male cats [9, 15]. In addition, with weight gain visceral and subcutaneous fat are reported to increase to the same extent in cats [15].

More recently, in human medicine and rodent models, the adipokines resistin and omentin have been receiving increased attention. Resistin has previously been shown to inhibit insulin-stimulated glucose uptake in skeletal muscle of rats [28], and elevated levels have been proposed as a cause of obesity-related insulin resistance in rodents and humans [26, 28, 29]. Higher resistin mRNA levels have been detected in the subcutaneous adipose tissue of obese, post-menopausal women; these levels also correlated positively with body mass index (BMI), serum resistin concentrations, and insulin resistance [29, 30]. Recent evidence indicates that resistin mRNA levels are higher in the adipose tissues of obese when compared to leans cats [30] but blood concentrations of resistin have not yet been evaluated.

In humans, the adipokine omentin has been shown to enhance insulin-regulated glucose uptake in vitro [31]. It has been found to have similar circulating blood levels as adiponectin and a similar mechanism of regulation has been proposed [31, 32]. Numerous studies in humans have found circulating plasma levels and visceral adipose gene expression of omentin to be negatively correlated with BMI, fasting insulin, and measures of homeostatic model assessment of insulin resistance (HOMA-IR) [31, 33–35]. Obese individuals and males have been shown to have decreased serum levels, independently of each other, and diabetics and those with impaired glucose regulation have lower omentin levels [32]. However, its specific role in systemic glucose metabolism, and its role in metabolic syndrome and T2DM remains unclear [14, 31, 36]. Omentin has not yet been evaluated in cats.

The objectives of this study were to investigate the effects of gender and body adiposity on insulin sensitivity and circulating concentrations of the adipokines leptin, adiponectin, resistin, and omentin in adult cats. We hypothesized that feline obesity is associated with sex- dependent changes in circulating leptin, resistin, insulin, and adiponectin.

## Results

### Animals

**Fig. 1:**
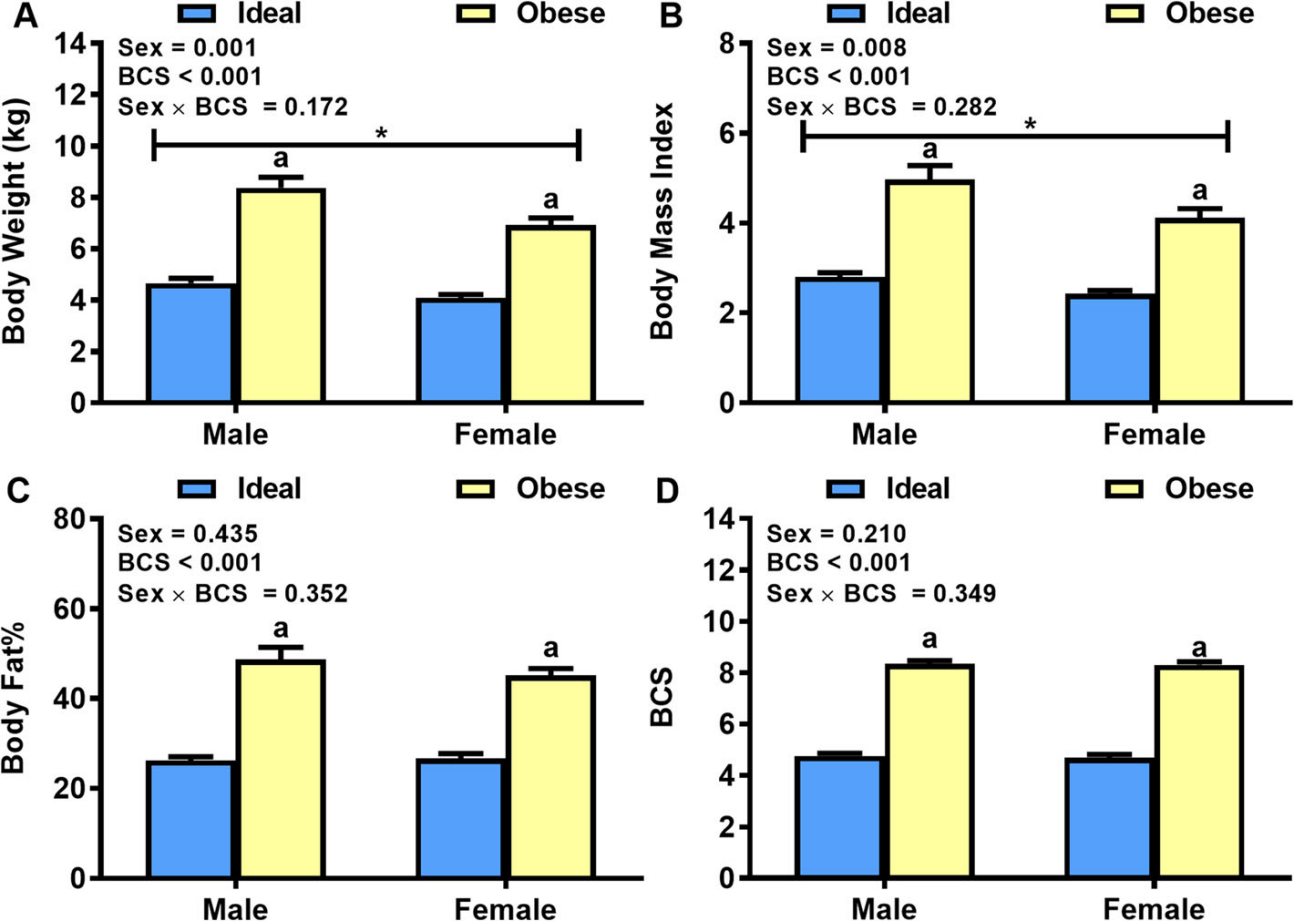
Body measurements. **a** Body weight and estimates of **b** Body Mass Index, **c** body fat%, and **d** body condition score (BCS), in a clinical population of ideal weight and obese cats of both genders (*n* = 16–17 /body condition/gender). Values are mean ± SE. ^a^*P* < 0.05, obese vs ideal weight . ^*^P < 0.05, male vs female

A total of 65 cats (age 6–9 years) were included in this study. As expected, the body weight, body condition score (BCS), BMI and body fat% of obese cats were 73, 79, 72 and 77% greater than ideal weight cats (*P* < 0.05), respectively (Fig. 1). Further, male cats had greater body weight and BMI than female cats, but with no sex differences in body fat% or BCS (*P* < 0.01).

### Metabolites

**Fig. 2:**
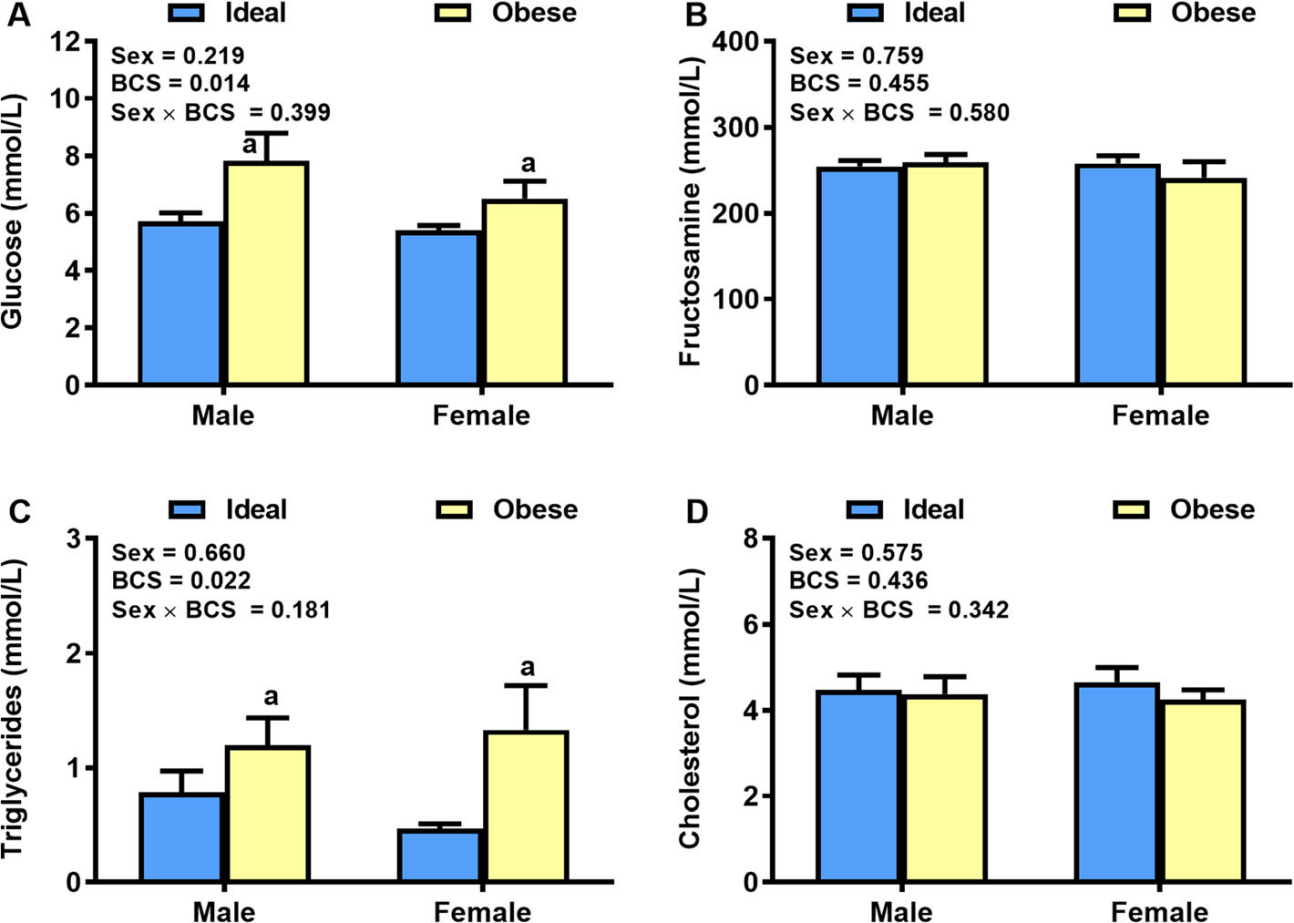
Plasma metabolite concentrations. Plasma concentrations of (**a**) Glucose, (**b**) Fructosamine, (**c**) Triglycerides and (**d**) Cholesterol in a clinical population of ideal weight and obese cats of both genders (*n* = 16–17/body condition/gender). Values are mean ± SE. ^a^*P* < 0.05, obese vs ideal weight

Fasting blood glucose and triglyceride concentrations were 30 and 143% greater in obese than ideal weight cats (*P* < 0.05), respectively, whereas, differences in serum fructosamine and cholesterol concentrations did not differ (Fig. 2). There were no gender differences in metabolites (*P* > 0.10).

### Plasma hormones

**Fig. 3:**
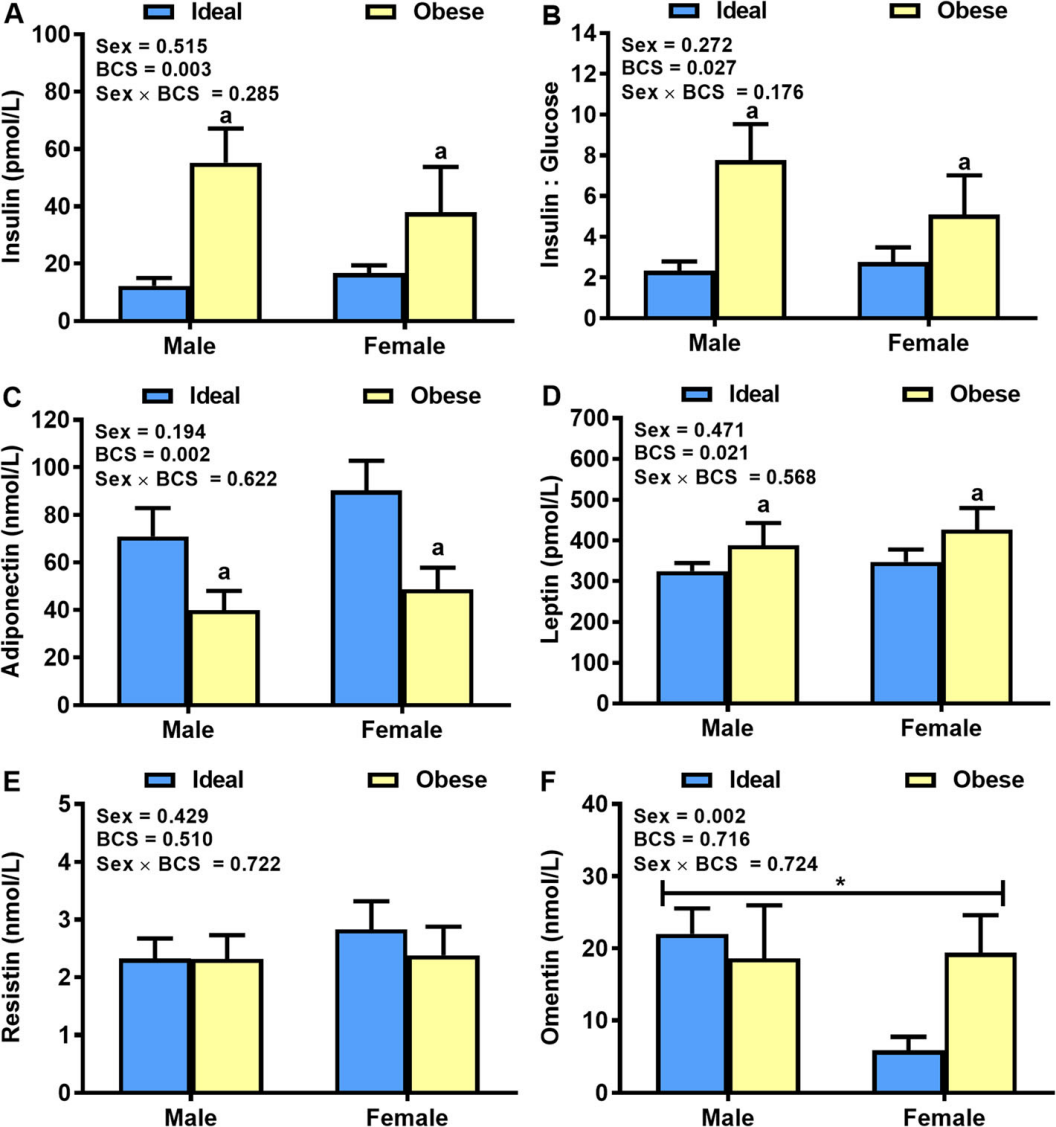
Plasma hormone concentrations. Plasma concentrations of (**a**) Insulin, (**b**) Adiponectin, (**c**) Leptin, (**d**) Resistin and (**e**) Omentin in a clinical population ideal weight and obese cats of both genders (*n* = 9–10/body condition and gender for insulin, adiponectin, leptin, and omentin; *n* = 16–17/body condition and gender for resistin). Values are mean ± SE. ^a^*P* < 0.05, obese vs ideal weight; ^*^P < 0.05, male vs female

The main effect of BCS was significant (*P* < 0.01) for plasma insulin, whereas, sex (*P* = 0.51) and sex × BCS interactions (*P* = 0.28) were not significant. The obese cats had 222% greater insulin concentrations than ideal weight cats (Fig. 3a). Further, obese cats had greater insulin: glucose ratio (*P* < 0.05) than ideal weight cats (Fig. 3b). There was a significant main effect of BCS (*P* < 0.01) for plasma adiponectin (Fig. 3c), whereas sex (*P* = 0.19) and sex × BCS interactions (*P* = 0.62) were not significant. Ideal weight cats had 46% greater adiponectin concentrations than obese cats. For plasma leptin concentrations, the main effect of BCS was significant (*P* < 0.05), whereas, sex (*P* = 0.47) and sex × BCS interactions (*P* = 0.28) were not. Obese cats had 32% greater leptin concentrations than ideal weight cats (Fig. 3d). Plasma resistin concentrations did not differ between ideal weight and obese cats (*P* = 0.51), or between genders (*P* = 0.43) (Fig. 3e). Plasma omentin concentrations did not differ between ideal weight and obese cats (*P* = 0.71). However, male cats had 56% greater omentin concentrations (*P* < 0.05) than female cats (Fig. 3f).

### Principal components and regression analyses

**Fig. 4:**
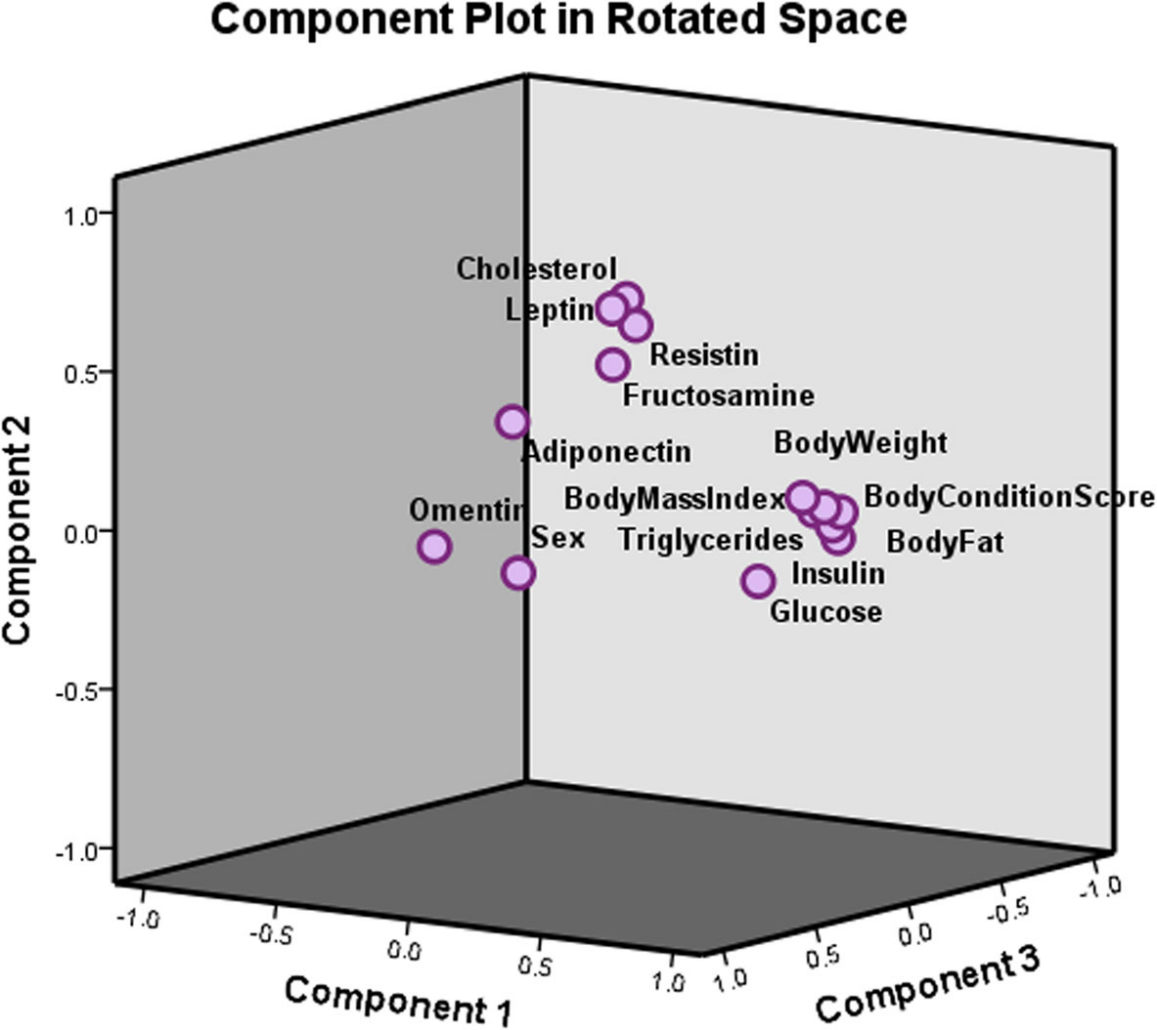
Principal Component Analysis of obesity-related indices, plasma metabolites and hormones in a clinical population of ideal weight and obese cats of both genders (*n* = 9–10/body condition and gender)

**Fig. 5:**
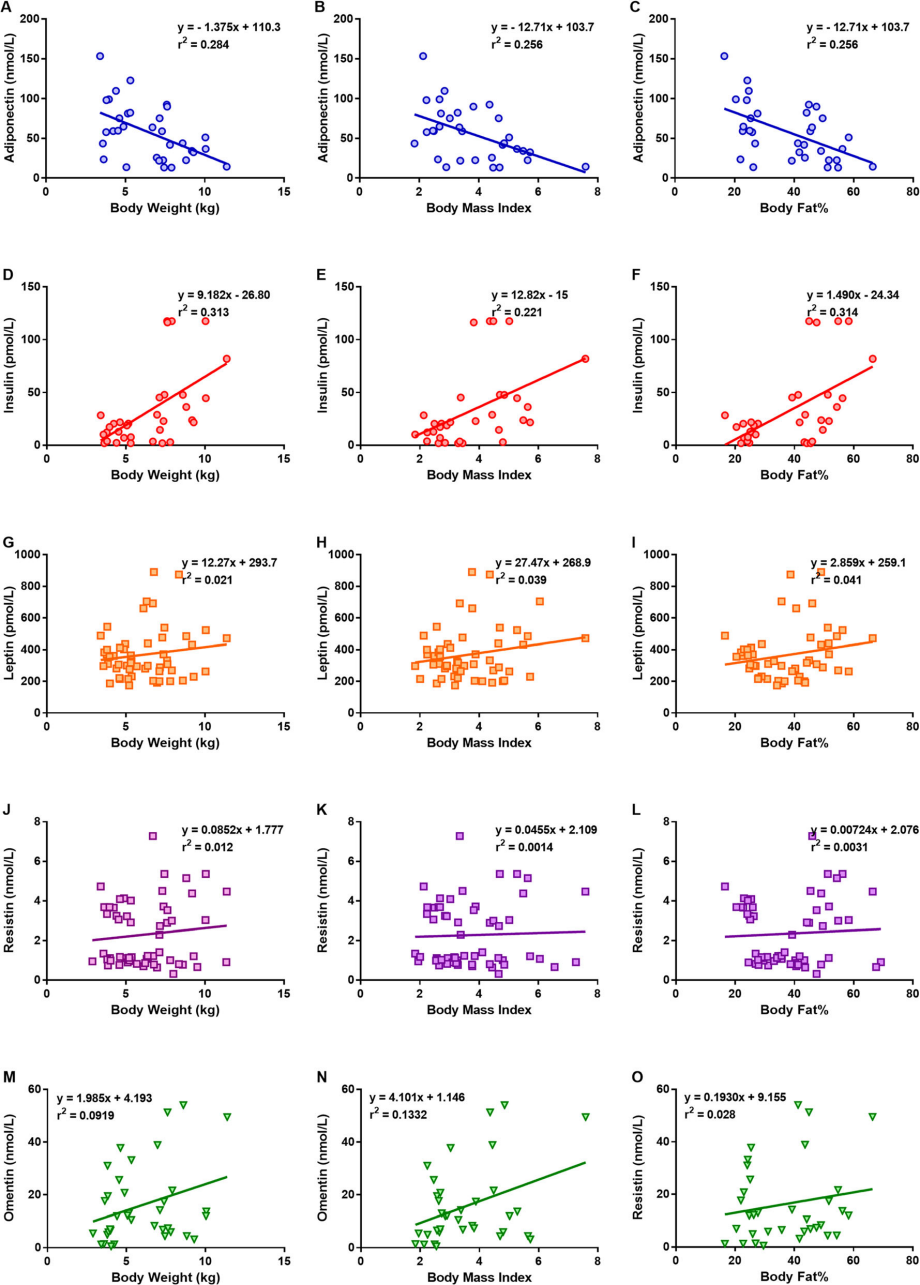
Linear regression of plasma concentrations of (A-C) adiponectin, (D-F) insulin, (G-I) leptin, (J-L) resistin and (M-O) omentin in cats

Principal component analyses (PCA) revealed that components 1, 2 and 3 each contributed to 32.33%, 11.81 and 10.03%, or 54.17% of the total variance, with rotated sums of squares loadings of 4.49, 1.59 and 1.52, respectively. As shown in Fig. 4, body weight (0.92), body fat% (0.90), BCS (0.88), BMI (0.87), insulin (0.64), triglycerides (0.59), glucose (0.46) and adiponectin (− 0.53) loaded heavily on the first component which likely reflects the obese status. This is further supported by the positive regression (*r*^2^ = 0.221 to 0.314) of insulin on body measures (body weight, BMI, body fat%), negative regression (*r*^2^ = 0.284 to 0.256) of adiponectin on body measures, and very weak regression of leptin (*r*^2^ = 0.021–0.041), resistin (*r*^2^ = 0.0014 to 0.012) and omentin (*r*^2^ = 0.028 to 0.1332), on the body measures (Fig. 5). Further, leptin (0.69), cholesterol (0.65), resistin (0.57) and fructosamine (0.49) loaded on the second component, which may be suggestive of diabetic predisposition. Omentin (0.78) and sex (0.71) loaded on the third component, which is suggestive of gender specific grouping.

## Discussion

Feline diabetes and obesity closely model these conditions in humans [37–39]. Adipokines are well known to play an important role in the pathophysiology of human diabetes and obesity [40, 41]; however, less is known about adipokines in felines. Here, we examined whether circulating concentrations of adipokines differ between healthy, mature ideal weight and obese cats, in a narrow age range, and whether such differences are influenced by gender. A major finding from our study is that obese cats are characterized by greater circulating concentrations of glucose, triglycerides, insulin and leptin, but lower adiponectin concentrations, than ideal weight cats, with no gender differences in these circulating markers. To our knowledge, this is the first study evaluating omentin in cats, and interestingly, we found that circulating omentin concentrations were greater in male than female cats but dissociated from body adiposity.

In this study obese cats had significantly higher fasting insulin and blood glucose concentrations when compared to ideal weight cats. Our results are not unexpected as previous studies support our finding with increased fasting blood glucose and insulin concentrations in obese versus lean cats [42–45]. Further, we also note that insulin and glucose were strongly associated with BCS, BMI and body fat %, with obese cats having a statistically greater insulin: glucose ratio than ideal weight cats. Blood collection was performed on unsedated animals. It is possible that the stress of sampling may have caused a physiologic and transient increase in blood glucose concentrations. An increase in insulin: glucose ratio has been shown to correlate to decreased insulin sensitivity in cats [44]. The finding of increased fasting insulin and blood glucose is suggestive of an incomplete compensatory response for decreased insulin sensitivity in our obese group. Although previous studies in neutered male and female cats showed physiological differences in glucose homeostasis between sexes [5, 20, 46], in our study population, no sex differences were noted for glucose, insulin, or insulin:glucose ratio. It is possible, however, that more sensitive measures of insulin sensitivity such as the hyperglycemic clamp, or oral glucose tolerance tests, may have revealed differences between sexes. Our study was consistent with other studies that found circulating concentrations of leptin to be positively correlated with weight but not gender [15–18, 21, 47]. Conversely, plasma concentrations of total adiponectin were decreased in obese cats with a significant negative association with adiposity measures in our study, which is in agreement with most [15, 19, 21, 47, 48], but not all [17, 23], studies in cats. We previously reported that adiponectin concentrations in newly diagnosed diabetic overweight cats were lower than lean controls [21]. Although the majority of diabetic cats in that study were male, reflecting the natural occurrence of disease, it was not designed to look at sex differences [21]. One study reported that male cats have reduced circulating concentrations of total adiponectin as well as high and low molecular forms of adiponectin, although adiponectin concentrations did not differ between lean and obese groups [17]. In the current study, there were no significant sex differences in circulating adiponectin concentrations.

In cats, there is conflicting evidence for whether increasing BCS is associated with a shift from subcutaneous to visceral fat [49]. It must be noted, however, that Hoenig et al. [15] found that in a population of lean and obese cats, with obesity induced by ad libitum feeding, there was no difference in fat distribution between subcutaneous and abdominal depots in males and females. Conversely, Okada et al. [49] found that in client-owned cats, increasing BCS was associated with a shift from subcutaneous to visceral fat. In the current study, we did not perform magnetic resonance imaging and dual-energy X-ray absorptiometry, and hence, the location of fat depots was unknown. Though some studies reported that visceral adipose tissue appears to have greater adiponectin mRNA expression [50, 51] and lower leptin transcript abdundance [52] than subcutaneous adipose tissue, others [30] have failed to detect such depot-specific differences. Concurrent evaluation of visceral and adipose mRNA expression and serum concentrations of these adipokines together direct quantification of adipose depots in adult obese and lean, male and female cats would be of interest.

For the first time we attempted to measure circulating concentrations of resistin and omentin in cats. Though a commercially-available feline specific ELISA kit was used to evaluate serum resistin levels, the performance of the assay was poor, with low recoveries and poor parallelism. Hence, no definitive conclusions could be drawn regarding the effect of obesity or sex on circulating resistin concentrations in cats. However, the greater resistin mRNA abundance in the subcutaneous and visceral adipose tissues of obese versus lean cats in one study [30] suggests that a difference in circulating concentrations may be present and warrants further investigation with alternative techniques.

In contrast to resistin, the assay performance was acceptable for measuring omentin in cats. Though circulating omentin concentrations did not differ between lean and obese cats, there was a clear sex difference with females having significantly lower levels and greater variation than males. All animals in this study were spayed or neutered prior to 6 months of age. It is unknown if early exposure to sex hormones could result in the sex difference noted for omentin concentrations later in life. Though fat distribution was not evaluated in the current study, whether sex differences in fat distribution contribute to the observed sexual dimorphism in omentin concentrations remains to be determined. Okada et al. [49] reported that, similar to humans, in naturally obese cats, fat deposition may be affected by BCS, and this could potentially explain the sex difference seen in serum omentin concentrations in the current study. In humans, the relationship of gender and omentin levels is unclear with one study reporting greater circulating omentin concentrations in women than men [34] and another finding higher omentin concentrations in men than women [53]. However, in contrast to our study, plasma omentin concentrations were negatively associated with obesity [34] and increased with weight loss in both men and women [53]. In the current study, and contrary to human studies, there were no correlations between omentin levels and body condition score, insulin, or glucose levels. Thus, it appears that the regulation, production and secretion of omentin in cats may be different than in humans. Another possibility for the gender difference seen is related to its production. Despite being an adipokine, omentin production in humans differs from other adipokines in two major ways. Firstly, it is produced by the stromal-vascular fraction of visceral adipose tissue as opposed to adipocytes and secondly, it is expressed in only very low quantities in subcutaneous adipose tissues [31]. Further studies evaluating adipose concentrations of omentin in cats and plasma omentin concentrations in male and female diabetic cats are warranted.

## Conclusion

In summary, we demonstrate that circulating concentrations of glucose, triglycerides, insulin and leptin are greater in obese than ideal weight cats, with reciprocal changes in adiponectin concentrations. Notably, circulating omentin concentrations exhibited sexual dimorphism and were greater in male than female cats. The underlying causative mechanisms for such differential effects of body adiposity and gender on circulating adipokines together with concurrent evaluation of gene and protein abundance in fat depots in cats warrants further research. Together these findings suggest that in healthy adult cats, body weight and adipose reserves are a more important determinant of circulating concentrations of the evaluated adipokines than gender.

## Methods

### Animals

Sixty-five cats were recruited and used in this study. Animal use was approved by the University of Calgary Veterinary Science Animal Care Committee (protocol# AC17–0187) and the Western College of Veterinary Medicine (WCVM protocol #20170106) Animal Research Ethics Board. Participating cats were recruited from faculty, staff, students, and clients of the hospital via word of mouth, email communications, and posters. Informed consent was obtained from the clients and owners. Inclusion criteria were ages of 5–9 years of age, neutered or spayed status, healthy to the owner’s knowledge and based on screening laboratory work that included packed cell volume (PCV), total solids (TS), serum biochemistry and total thyroxine (TT4) concentration, and body condition scores of 4–5 (ideal or lean) or 8–9 (obese) on a 9-point scale [54, 55]. Exclusion criteria included any chronic or ongoing condition including gastrointestinal disease, hyperthyroidism, diabetes mellitus, or current or recent treatment with drugs known to influence glucose homeostasis.

Cats were classified as ideal (BCS of 4 or 5/9) or obese (BCS 8 or 9/9) based on a standard 9-point body condition scoring (BCS) system. Tape measures and digital scales were used to take morphometric body measurements and weigh each animal. Previously reported techniques to assess body mass index (BMI) and body fat percentage were used to best ensure differences between study groups in the absence of use of DEXA or MRI. Body mass index (BMI) was calculated using the following calculation: BMI = body weight (kg)/ (body length [meters]) × height [meters]) as previously described [9, 56]. Body fat percentage was assessed with the previously reported equation: Percentage Body fat = [(([RC/0.7062]-LIM)/0.91560)-LIM] [42, 57], where RC was the circumference of the rib cage (cm) and LIM was the length of the lower limb from the middle of the patella to the dorsal hip (cm) .

### Sampling

Sampling took place at the WCVM Teaching Hospital or the University of Calgary Faculty of Veterinary Medicine (UCVM) Clinical Skills Building or clinics that belong to the UCVM Distributed Veterinary Learning Community from October 2017 to February 2018. All sampling and measurements were performed by 1 or more of the researchers (MW, CM [UCVM]; KT, ES, CM [WCVM]). Cats were fasted for 12–18 h prior to sampling.

Blood collection was performed on un-sedated animals, Once collected, blood was placed into serum tubes and EDTA-containing tubes for plasma and was kept on ice between sampling and processing. Blood samples for serum were allowed to clot and were centrifuged within 20 min of blood collection. An aliquot of serum from each cat was used to determine a biochemical profile, TT4, and serum fructosamine, using standard laboratory methods at a commercial laboratory (Antech Diagnostics, Calgary, AB). Following blood collection in EDTA tubes, plasma was separated following centrifugation within 20 min of collection, aliquoted into multiple tubes, and then stored at − 80 °C until analysis. A history and lifestyle questionnaire was completed by all owners at the time of sampling which included information on recent weight changes, diet, age of spay/neuter, and medical history.

### Measurement of plasma hormone and serum metabolite concentrations

Fasting plasma concentrations of insulin, adiponectin, leptin, resistin and omentin were analyzed using commercially available enzyme linked immunosorbent assay (ELISA) kits. Based on our previous study on lean and obese cats [21], in a randomized design with α = 0.05, the effect size, SD, power and sample size for plasma leptin concentrations were 0.75, 8.62, 81% and 7, and for adiponectin were 0.72, 9.03, 82% and 9, respectively. Hence, a minimum sample size of 9 for each body condition and gender was selected for hormone assays. Each sample was assayed in duplicate following the manufacturer’s recommended protocols. All assays underwent validation procedures using pooled cat plasma. To minimize the effect of inter-assay variability, the samples were distributed, as necessary, so that each plate received an approximately equal number of samples from each treatment group. Further, separate plasma aliquots were thawed and used for assay of each analyte, and if repeat runs were necessary the plasma was freeze-thawed for a maximum of two times. Inter-assay coefficient of variation (CV) for some hormones was assessed by running pooled cat plasma in duplicate on each plate. Assay sensitivity was defined as the lowest detectable concentration of a hormone from all the samples analyzed. Fasting serum metabolite concentrations (glucose, triglycerides, cholesterol, and fructosamine) were measured using standard laboratory protocols at a commercial laboratory (Antech Diagnostics, Calgary, AB).

Insulin (*n* = 38) was measured using a feline specific assay that we validated and reported on in cats previously [58]. The assay has a range of 1.5–683 nmol/L (10–1233-01, Mercodia, Uppsala, Sweden). All samples were run in a single assay, and the intra-assay CV was 13%, assay sensitivity was 2.36 pmol/L, and a spike of 25 pmol/L of feline insulin to pooled cat plasma resulted in 119% recovery.

Leptin (*n* = 38) was measured using a feline leptin-specific assay with a range of 0–125 nmol/L (MBS057075, MyBioSource Inc., San Diego, CA). The intra-assay and inter-assay CV’s were 5 and 35%, respectively, and the assay sensitivity was 171 pmol/L. Spikes of 62.5, 250 and 500 pmol/L of feline leptin in pooled cat plasma yielded recoveries of 71, 62 and 72%, respectively. Linear regression of expected versus measured concentrations for leptin from serially diluted pooled plasma (1:2 to 1:4) yielded a slope of 0.98, r^2^ of 0.96, and Y-intercept of 0.085.

Adiponectin (*n* = 38) was measured as we [21] and others [47] reported previously, with a human adiponectin assay that has a range of 3–694 nmol/L (RD191023100, BioVendor, Brno, Czech Republic). All samples were run in a single assay, and the intra-assay CV was 4%, and assay sensitivity was 13.17 nmol/L. Linear regression of expected versus measured concentrations for adiponectin from diluted pooled plasma yielded a slope of 1.52, r^2^ value of 1 and Y-intercept of − 4.53.

Resistin (*n* = 65) was measured using a feline resistin-specific assay with a range of 0.25–8 nmol/L (MBS03076, MyBioSource Inc., San Diego, CA). The intra- and inter-assay CV’s were 6 and 15%, respectively, and the assay sensitivity was 0.316 nmol/L. Spikes of 0.5, 1, 2 and 4 nmol/L of feline resistin in pooled cat plasma yielded recoveries of 50, 55, 46 and 47%, respectively. Linear regression of expected versus measured concentrations for resistin from serially diluted pooled plasma (1:2 to 1:4) yielded a slope of 0.95, r^2^ values of 0.98, and Y-intercept of 0.74.

Omentin (*n* = 39) was measured using a feline specific assay with a range of 0.89–57 nmol/L (FEE0035, Biotang Inc., Lexington, MA). The intra-assay and inter-assay CV’s were 12 and 23%, respectively, and the assay sensitivity was 1.25 nmol/L. Spikes of 0.89, 3.57 and 28.57 nmol/L of feline omentin in pooled cat plasma yielded recoveries of 118, 77 and 101%, respectively. Linear regression of expected versus measured concentrations for omentin from serially diluted pooled plasma (1:2, 1:4, 1:8) yielded a slope of 0.75, r^2^ values of 0.85, and Y-intercept of 2.39.

### Statistics

Data were analyzed using IBM SPSS® v20 (New York, USA). The data for hormones, metabolites and body morphometrics measurements were analyzed by using gender, body condition (ie. obese vs ideal weight) and gender × body condition interaction in a general linear model, followed by Tukey’s *post-hoc* separation of means. Prior to analyses, data were log or square root transformed as necessary to improve normality. Principal Component Analysis (PCA) was used to reduce dimensions in the dataset. Following the Barlett test of sphericity (*P* < 0.01) and Kasier-Meyer-Olkin Measures of Sampling Adequacy (0.708), four major components were initially examined each with an eigenvalue greater than 1. Since two of the components were correlated (*r* = 0.27), only three major components were extracted following Direct Oblimin rotation. To assess the relationship between the body morphometric measurements and plasma hormones, a linear regression analyses was done. Data are reported as mean ± SE. Significance was set at *P* ≤ 0.05.

## Data Availability

The datasets used and/or analyzed during the current study is available from the corresponding authors on reasonable request.

## Abbreviations

BCS: Body Condition Score
BMI: Body Mass Index
CV: Coefficient of Variation
DM: Diabetes Mellitus
ELISA: Enzyme Linked Immunosorbent Assay
HOMA-IR: Homeostatic Model Assessment of Insulin Resistance
IR: Insulin Resistance
LIM: Length of the Lower Limb
PCA: Principal Component Analysis
PCV: Packed Cell Volume
RC: Rib Cage
TS: Total Solids
TT4: Total Thyroxine
Type 2 DM: Type 2 Diabetes Mellitus
UCVM: University of Calgary Faculty of Veterinary Medicine
WCVM: Western College of Veterinary Medicine
